# Modelling methicillin-resistant *Staphylococcus aureus* decolonization: interactions between body sites and the impact of site-specific clearance

**DOI:** 10.1098/rsif.2021.0916

**Published:** 2022-06-15

**Authors:** Onur Poyraz, Mohamad R. A. Sater, Loren G. Miller, James A. McKinnell, Susan S. Huang, Yonatan H. Grad, Pekka Marttinen

**Affiliations:** ^1^ Department of Computer Science, Aalto University School of Science, Aalto, Finland; ^2^ Department of Immunology and Infectious Diseases, Harvard TH Chan School of Public Health, Boston, MA, USA; ^3^ Division of Infectious Diseases, Lundquist Institute at Harbor-UCLA Medical Center, Los Angeles, CA, USA; ^4^ Division of Infectious Diseases, University of California Irvine School of Medicine, Irvine, CA, USA

**Keywords:** methicillin-resistant *Staphylococcus aureus*, decolonization protocol, machine learning, coupled hidden Markov model, Markov chain Monte Carlo

## Abstract

Methicillin-resistant *Staphylococcus aureus* (MRSA) can colonize multiple body sites, and carriage is a risk factor for infection. Successful decolonization protocols reduce disease incidence; however, multiple protocols exist, comprising diverse therapies targeting multiple body sites, and the optimal protocol is unclear. Standard methods cannot infer the impact of site-specific components on successful decolonization. Here, we formulate a Bayesian coupled hidden Markov model, which estimates interactions between body sites, quantifies the contribution of each therapy to successful decolonization, and enables predictions of the efficacy of therapy combinations. We applied the model to longitudinal data from a randomized controlled trial (RCT) of an MRSA decolonization protocol consisting of chlorhexidine body and mouthwash and nasal mupirocin. Our findings (i) confirmed nares as a central hub for MRSA colonization and nasal mupirocin as the most crucial therapy and (ii) demonstrated all components contributed significantly to the efficacy of the protocol and the protocol reduced self-inoculation. Finally, we assessed the impact of hypothetical therapy improvements *in silico* and found that enhancing MRSA clearance at the skin would yield the largest gains. This study demonstrates the use of advanced modelling to go beyond what is typically achieved by RCTs, enabling evidence-based decision-making to streamline clinical protocols.

## 1. Introduction

Methicillin-resistant *Staphylococcus aureus* (MRSA) is a common antimicrobial-resistant pathogen in community and healthcare settings [1,2], causing an estimated 320 000 infections in hospitalized patients and over 10 000 deaths in the USA in 2017 [3]. Progress in reducing invasive MRSA infections has slowed, underscoring the importance of continued innovation and effort to prevent disease [4]. As MRSA carriage is a major risk factor for invasive disease, efforts at prevention centre on the promotion of decolonization protocols and body hygiene as well as environmental cleaning [5]. The most common *S. aureus* carriage site is the anterior nares, but MRSA can also colonize the perineum and groin, the axilla, the pharynx, as well as other body sites [6,7]. While the anterior nares have been identified as a key reservoir for transmission, and nasal colonization is a major risk factor for invasive disease [8], the extent of interaction among colonization sites and the importance of additional decolonization products targeting other body sites, and the value of increasing their adherence in decolonization protocols remain unclear. It would be ideal to understand the interactions between body sites and the attributable effect of each therapy on overall body clearance. For example, if carriage at a particular body site appears to be dependent on carriage at other body sites, therapies targeting the influencing site would be more efficient, while therapies targeting the dependent site might be less effective for reducing the total body carriage. Achieving this goal requires a detailed understanding of the dynamic relationships of colonization between and among sites.

The Changing Lives by Eradicating Antibiotic Resistance (CLEAR) trial demonstrated that the use of a post-discharge decolonization protocol in MRSA carriers reduces infection and hospitalization rates [9]. In the trial, 2121 study participants were randomized into two groups to test the impact of the decolonization protocol: the *education* group (*n* = 1063) received an educational binder on hygiene, cleanliness and MRSA transmission; the *decolonization* group (*n* = 1058) received the same information and as well underwent decolonization protocol for 5 days twice monthly for six months, with the protocol consisting of nasal mupirocin and chlorhexidine body and mouth wash. During these six months, swabs were collected from the participants at discharge from the hospitalization and three follow-up visits, which approximately took place at months 1, 3 and 6 after the discharge. Samples were taken from the nares, skin (axilla/groin), throat and, if present, any wound. Participants had different numbers of observations because of trial exits or skipped visits. Overall, 20 506 samples (10 464 and 10 042 from the education and decolonization groups, respectively) were received in the first six months of follow-up. Additionally, the dataset included reported adherence to the protocol, enabling the assessment of both real-world uptake and consideration of the ideal scenario of full compliance.

**Figure 1.**
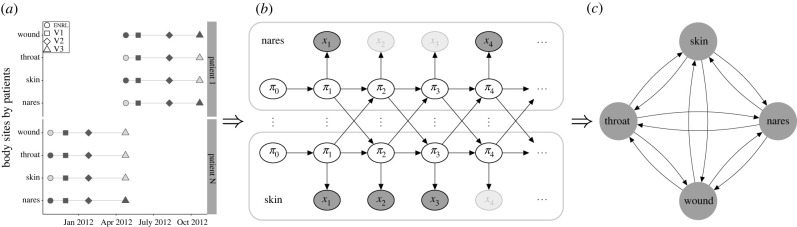
Overview of the modelling strategy. (*a*) Visit records, (*b*) illustration of the coupled hidden Markov model (CHMM) and (*c*) estimated interactions between body sites. (*a*) An example with visit records for two participants. Evaluated visits approximately took place in one (V1), three (V2) and six (V3) months after enrol ment (ENRL) in the trial. Filled markers correspond to the collected swabs, and faded markers represent samples missing due to trial exits or skipped visits [9]. (*b*) Here, for clarity, the CHMM is illustrated with two (nares and skin) of the four body sites (nares, skin, throat and wound). In practice, the model includes all four sites. *π*_*t*_ represents the unobserved true states (whether the site was colonized or not), and *x*_*t*_ represents observed states (whether MRSA was detected from the swab or not) at time *t*. The faded observation nodes correspond to missing observations, which are straightforward to analyse with the CHMM. The sequential model can be used to predict the dynamics of carriage. (*c*) The CHMM allows us to estimate and visualize interaction dynamics graphically, where edges represent the strength and direction of the interaction.

In this paper, our goal was to model the process of MRSA carriage, with and without the decolonization protocol. Successful modelling can enable characterization of the interactions among MRSA colonization at different body sites and the efficiency of each protocol component. Using this information, we can predict how the decolonization protocol could be more efficient. To achieve these goals, we used a coupled hidden Markov model (CHMM [10–16], an extension of the standard hidden Markov model, HMM [17–19]), where the probability of colonization at a particular body site in the next step depends not only on the colonization of the same site but also on the colonization of the other body sites (figure 1). We developed a novel formulation of the CHMM: *Additive-CHMM,* where the probability of colonization at a particular site is an additive function of colonization at the other sites. According to a predictive model selection criterion (leave-one-out cross-validation [20]), the Additive-CHMM had superior accuracy compared with a set of site-specific standard HMMs and other formulations that we developed (see electronic supplementary material, table S1). Consequently, the outcomes of the Additive-CHMM are presented and discussed in the main text. Models are described in the §4 and their advantages and disadvantages are discussed in detail in the electronic supplementary material. We provide a practical application programming interface (API) as an R-package that implements these models with an efficient Metropolis-within-Gibbs Markov chain Monte Carlo (MCMC) algorithm, which yields Bayesian credible intervals (CI) for all model parameters (see §4).

## 2. Results

**Figure 2.**
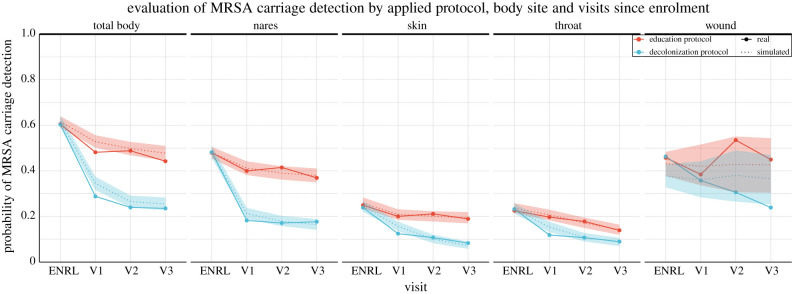
The observed decrease in MRSA carriage detection over time by body site and study arm compared with the decrease predicted by the model. In the trial, study subjects were in the education group or the decolonization group, which consisted of applying mupirocin to the nares, chlorhexidine mouthwash (CHG Oral) to the throat and chlorhexidine body washes (CHG Skin) to the skin and, if present, wound. The figure shows site-specific and total-body carriage probabilities and clearance rates in the two groups, along with model predictions. We note that the number of samples from wounds was relatively small, which yielded larger uncertainty in the wound-associated estimates. Visits approximately took place in one (V1), three (V2) and six (V3) months after enrol ment (ENRL, i.e. hospital discharge) in the trial. Dotted lines and shaded regions represent the mean and 90% credible intervals (CI) of the model predictions, and the dots connected by the solid lines represent values observed in the data.

The CHMM accurately predicted the decrease in MRSA carriage over the study period. The key metric of success for our model was the extent to which it recapitulates the clearance in MRSA carriage over the study period. To examine this posterior predictive checking, we (i) estimated the parameters of the CHMM in both education and decolonization groups, then (ii) simulated patient trajectories using parameters from the estimated models, and finally (iii) compared the reduction in colonization in our model-based simulations with the observed changes in the CLEAR trial data. The predicted reduction of the carriage for individuals was from 61% (90% CI is 59–63%) to 47% (44–50%) in the education group and down to 25% (22–28%) in the decolonization group, which accurately matched the observations (85% of the observations fell inside the 90% CIs, figure 2). Moreover, the predicted reduction at any single site was accurate as well. To further validate the model’s ability to capture the dynamics of MRSA carriage, we (i) applied cross-validation (CV) test to inspect the predictive performance (see electronic supplementary material, figure S1) and (ii) tested the model using synthetic data to inspect the ability to recover the true parameters and the impact of errors on the model predictions (see electronic supplementary material, figure S2).

**Figure 3.**
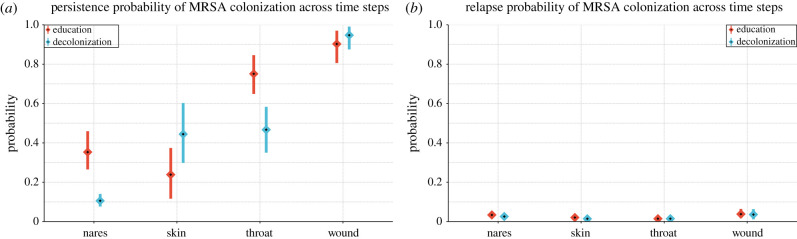
Estimated (*a*) persistence and (*b*) relapse probabilities of MRSA colonization by body site. (*a*) *Persistence probability* is defined as the probability that a site will be colonized in the next time step, given it was colonized in the previous time step while other sites were not colonized. (*b*) *Relapse probability* is defined as the probability that a site will be colonized in the next time step, given it was not colonized in the previous time step while other sites also were not colonized. The calculation of the posterior distributions is explained in the §4. Means and 90% credible intervals (CI) are represented in the figure by diamonds and lines, respectively.

The decolonization protocol decreased the persistence of MRSA colonization in the nares and throat independently of other sites. First, we wanted to study the impact of the full decolonization protocol on each particular site while not accounting for any dependencies of carriage between sites (i.e. sites were evaluated in isolation). To do this, we estimated the persistence of colonization for each body site by calculating the probability that the site would be colonized in the next time step, given it was colonized in the previous time step and other sites were not colonized. We found that decolonization protocol significantly reduced the persistence at the nares from 35% (26–45%) to 10% (7–14%), and throat from 75% (64–84%) to 47% (35–58%), while the persistence of colonization at skin and wound were not significantly affected when these sites were evaluated in isolation (figure 3*a*) [21]. We also estimated the relapse probability, i.e. the probability of a site getting colonized given it was not colonized in the previous time step and other sites also were not colonized. These probabilities were small and did not seem affected by the decolonization protocol (figure 3*b*). We note that the number of samples from wounds was relatively small, which yielded larger uncertainty in the wound-associated parameters. Repeating the analysis only on patients who reported adherence to the protocol led to almost identical results since most (70%) of the data were collected from adherent patients (see electronic supplementary material, figure S6). The model can also estimate other valuable parameters, for example, the sensitivity and specificity of the detection of MRSA in a swab, as those are immediately available as the emission parameters of the CHMM (see electronic supplementary material, figure S4).

**Figure 4.**
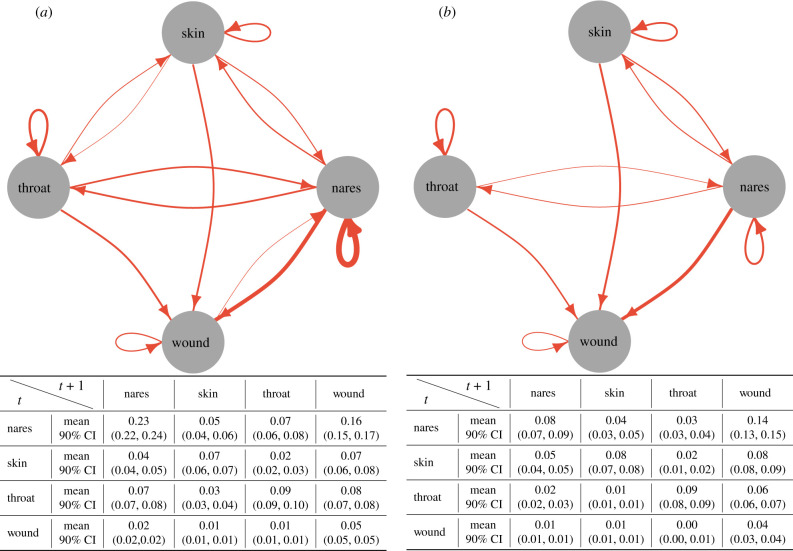
The amount of MRSA transmission among body sites in (*a*) education and (*b*) decolonization groups. The edges in the graphs show the estimated proportion of patients with the given transmission between body sites in a time step (corresponding to one month). They are estimated by scaling the transmission probabilities (see electronic supplementary material, figure S5) with the observed proportion of patients colonized in the source site of the transmission. Edges were excluded from the graph if the expected proportion was lower than 0.02. The edge thickness represents the expected value, and the tables show the means and the respective 90% CIs for all relations.

The decolonization protocol reduced the transmission of MRSA between body sites. We quantified the interactions between body sites in two ways: first, we estimated the probability of MRSA transmission within a time step (corresponding to one month) from the source site to the target (see electronic supplementary material, figure S5) conditional on only the source being colonized, by simulating 1000 datasets from the estimated model. Second, we estimated the proportion of patients with a given MRSA transmission between body sites (figure 4), which scales the former transmission probabilities with the observed proportion of patients colonized in the source site of the transmission. First, we found that the most critical determinant of colonization at any site was whether the site itself was colonized in the previous time step. Second, the nares acted both as a source and a sink for transmission, highlighting its role as a hub for MRSA colonization. Third, the strength of most dependencies decreased significantly by the decolonization protocol. These findings, therefore, indicate two mechanisms responsible for the efficiency of the decolonization protocol: (i) the protocol decreased persistence in the nares, which is a central accumulation hub for colonization (figure 3) and (ii) the protocol weakened links between body sites, reducing self-inoculation (figure 4).

**Figure 5.**
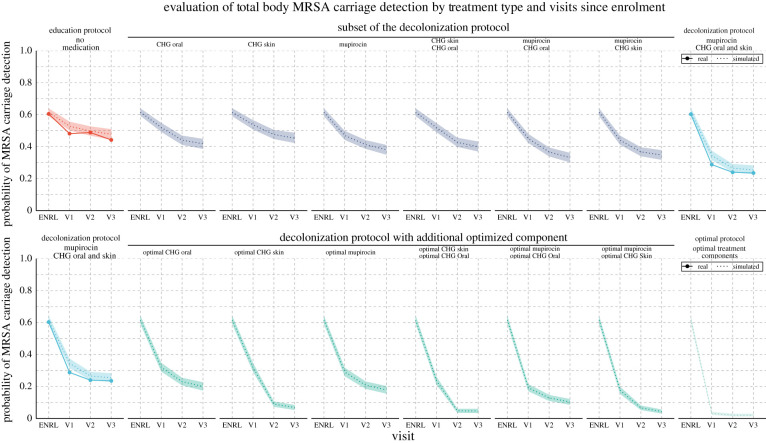
The observed decrease in MRSA carriage detection over time compared with the decrease predicted by the model for actual and hypothetical therapies. In the trial, study subjects were in the education group or the decolonization group, which consisted of applying mupirocin to the nares, chlorhexidine mouthwash (CHG Oral) to the throat and chlorhexidine bodywash (CHG Skin) to the skin and, if present, wounds. The predictions for hypothetical therapies assumed either that only a subset of medications was applied or that the decolonization protocol was used with an additional optimized intervention that resulted in immediate clearance of the corresponding target site. Visits approximately took place in one (V1), three (V2) and six (V3) months after enrolment (ENRL) in the trial. Dotted lines and shaded regions represent the mean and 90% credible intervals (CI) of model predictions, and the dots connected by the solid lines represent values observed in the data.

**Figure 6.**
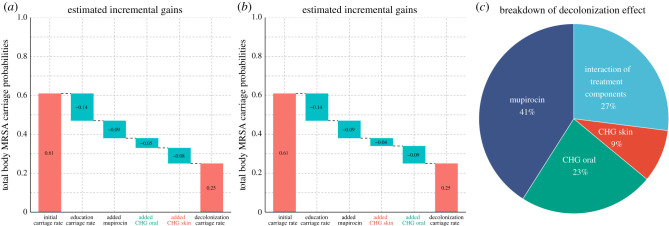
The estimated (*a*,*b*) incremental and (*c*) marginal contribution of each therapy on the efficiency of a decolonization protocol. In the trial, study subjects were in the education group or the decolonization group, which consisted of applying mupirocin to the nares, chlorhexidine mouthwash (CHG Oral) to the throat and chlorhexidine bodywash (CHG Skin) to the skin and, if present, wounds. (*a*,*b*) The plots show how the total body carriage at the end of the study decreases when different components are added to the protocol. The cumulative effect is shown for two different orders of adding components. (*a*) In the optimal order (where always the component that most increases the efficiency is added) has CHG Oral added before CHG Skin. (*b*) The order of adding these two components is reversed to show that the most recently added component seems better than it is because it creates an interaction effect between the added therapy and the therapies already included in the protocol. (*c*) The marginal effect of each therapy is calculated using applied single-site therapy on top of education protocol. Interaction of protocol components reflects additional gains achieved in the full decolonization protocol, which is not visible in the sum of marginal effects. The confidence intervals are already shown in figure 5 and written in the text.

Nasal mupirocin on the nares was estimated as the single most efficient therapy, but all therapies contributed to the efficiency of the decolonization protocol. The importance of the nares has been noted in a study in an intensive care unit [22]. However, the added value of decolonization efforts that focus on body sites other than the nares is not well understood. To quantify the impact of decolonization efforts at each individual site while accounting for interactions between body sites, we predicted the efficiency of hypothetical simplified protocols, consisting of only a subset of the therapies in the full decolonization protocol. Application of nasal mupirocin on the nares alone decreased the estimated total body carriage of MRSA from 61% to 38% (35–41%), compared with 25% (22–28%) of the full decolonization protocol and 47% (44–50%) of education protocol (figure 5). Other single therapies were inferred to be less effective than mupirocin, such that only using chlorhexidine mouthwash on the throat (CHG Oral) reduced total body carriage to 42% (39–44%) and only using chlorhexidine bodywash on the skin and wounds (CHG Skin) reduced total body carriage to 45% (42–48%), a minor improvement compared with the 47% (44–50%) of education protocol alone. When we inferred the anticipated success of combinations of two therapies, the best combinations always included mupirocin: CHG Skin + mupirocin decreased carriage to 34% (31–37%) and CHG Oral + mupirocin to 33% (30–36%). By contrast, the combination CHG Skin + CHG Oral, i.e. leaving out mupirocin, decreased carriage only to 39% (36–42%). These results show that all combinations of two therapies had lower efficiency than the full protocol indicating each therapy contributed to the full effect, even if no effect was seen on targeted sites when sites were evaluated in isolation (figure 3*a*). So, even though CHG Skin does not appear to affect the target site directly, it is still beneficial, and we hypothesize that the impact comes instead through reduced transmission between body sites (figure 4). Figure 6 shows (i) the incremental gain of adding therapies to the protocol and (ii) marginal effects of each therapy on the efficiency of the decolonization protocol. Results also indicate that the full effect is greater than the sum of marginal effects, demonstrating the synergy of applying therapies in combination.

Enhancing clearance at the skin was predicted to achieve the most significant gain in overall decolonization success. The key metric of success for decolonization is the extent to which it reduces MRSA carriage. We used our model to predict the impact of enhanced effects on the components of the decolonization protocol. We assumed optimal effects that resulted in immediate clearance of a specific site. This could be achieved either through a more effective replacement product, an additional product applied to the site on top of the existing protocol, or training to achieve the appropriate application of the protocol with high adherence leading to immediate clearance. The result reflected the relative inefficiency of the original CHG Skin therapy (figures 3*a* and 5), such that the most significant improvements were seen by improving this therapy. In particular, the estimated final carriage with the full protocol but optimized nasal mupirocin was 17% (15–20%), while optimizing the throat (CHG Oral) or skin/wound (CHG Skin) therapies decreased the carriage to 20% (17–22%) or 6% (5–8%), respectively. Optimizing therapies both on nares + the throat has the potential to reduce carriage further down to 10% (8–12%) compared with 4% (3–5%) of the nares + skin/wound, and 4% (3–6%) of the throat + skin/wound.

## 3. Discussion

We presented a model for the dynamic process of MRSA carriage that quantified interactions among colonization at different body sites, quantified the impact of each therapy, and predicted the decrease in MRSA carriage over the study period. We found that not all therapies contributed equally to successful decolonization. Among the CLEAR trial components, mupirocin was estimated as the most effective therapy; still, our results also indicated that all therapies were essential to the full effect of the complete protocol. Furthermore, our results indicated that enhancing the chlorhexidine bodywash therapy has the greatest potential for improving the effectiveness of the decolonization regimen.

As with all modelling, our analysis made several simplifying assumptions. First, the analysis focused on MRSA clearance on individuals, but the decolonization protocol may have other benefits, e.g. reduction in transmission between patients [23,24]. Second, we assumed that the missingness of observations (including trial exits, skipped visits or non-present wounds) did not depend on the colonization status of the body site, which is probably not always correct; for example, if a previously colonized wound is healed and not colonized anymore, no additional samples would be taken from the wound. Finally, in our optimal therapy analysis, we assumed that (i) all patients followed the same protocol regardless of their initial site of colonization, and (ii) the sensitivity and specificity of the swabs were not affected by the new optimal therapies. However, the importance of the therapies on these parameters has been noted in a previous study [25]. Also, it should be noted that the details of a decolonization protocol may have implications other than the impact on clearance. For example, they might (i) affect the cost of the protocol or adherence to it, or (ii) have other side-effects, e.g. altering the body’s microbiota. Modelling these effects is beyond the scope of the present work. The model could be improved further by (i) incorporating the type, the burden and initial probabilities of MRSA colonization because heterogeneity in treatment responses among patients, strain types of MRSA and the initial colonization distribution might affect the decolonization dynamics [26], and (ii) using semi-Markov approaches in the CHMM to explicitly model the variations in time intervals between the visits (currently the longer intervals were assumed to include missing in-between observations). Our analysis was based on a Bayesian approach, requiring a specification of priors on model parameters to express what is believed about their values before seeing the data. We used weakly informative priors in accordance with the literature [25], and the comparison included in the electronic supplementary material, table S1 shows that the model fit was not sensitive to the exact values of the priors. Despite the simplifications, the method successfully passed a comprehensive set of diagnostic tests for model fit and convergence of the algorithm.

Our analysis aims for infection control practitioners and researchers (i) to show the potential relative gains from the components of the decolonization protocol and (ii) to assist in understanding colonization dynamics and their interaction with the decolonization protocol. To this end, we provide modelling tools that may inform further clinical trials and practice, and we hope these tools help design even more effective decolonization protocols.

This study indicated that the impact of individual interacting components of complex clinical protocols and randomized controlled trial (RCT) data can be rigorously deconvoluted and assessed *in silico* by machine learning tools like those used here, thereby enabling the design of interventions that are more efficient, easier to adhere to, and more likely to be successful. This analytical approach thus demonstrates how data from RCTs can inform about biological processes as well as guide improvements in clinical protocols and decision-making.

## 4. Methods

### 4.1. Isolate collection

MRSA isolates were collected as part of the CLEAR trial. The trial was designed to compare the impact of a repeated decolonization protocol plus education on general hygiene and environmental cleaning with education alone on MRSA infection and hospitalization [9]. Study subjects in the trial were recruited from hospitalized patients based on an MRSA positive culture or surveillance swabs. After recruitment, swabs were obtained from different body parts of subjects (nares, skin, throat and wound) around the time of hospital discharge (ENRL) and at one, three, six and nine months (V1–V4, respectively) following the initial visit, after which the swabs were cultured on chromogenic agar. The application of decolonization protocol lasted only for six months, and consequently, we only modelled visits until V3 in this study. We note that some enrolled study subjects, despite a positive culture (clinical or surveillance) during the hospital stay, did not have discharge swabs positive for MRSA at the first time point (ENRL). The data used in this study are collected from people aged over 18 (average age = 56, s.d. = 17) for both the education and decolonization groups, with eligibility requirements also including hospitalization within previous 30 days and positive testing for MRSA during the enrolment hospitalization or within the 30 days before or afterwards. Exclusion criteria included hospice care and allergy to the decolonization products. Over the course of the trial, 98 of 1063 participants (9.2%) in the education group and in 67 of 1058 (6.3%) in the decolonization group developed MRSA infections, and 84.8% of the MRSA infections resulted in hospitalization. More details about recruitment and eligibility, follow-up, sample collection and data preprocessing can be found in [9].

### 4.2. Model

In this section, to describe the properties of CHMM, we initially described the properties of HMMs. Then, we presented the details of CHMMs, which were built on the HMM.

#### 4.2.1. Hidden Markov Model

Markov models are a well-known tool for time-series analysis. Hidden Markov models (HMM) are a special case where Markov process is observed indirectly through noisy observations. Discrete-time discrete-state hidden Markov model with a latent sequence ***π***, $\pi_{t}\in \{1,\ldots ,K\}$ and observations **x**, $x_{t}\in \{1,\ldots ,L\}$ for t∈{1,…,τ}, is defined as

4.1 $$p(\pi_{1:\tau },x_{1:\tau })=\underset{\text{initial state}}{\underbrace{\;p(\pi_{1})}}\left[\prod_{t=2}^{\tau }\underset{\text{transition}}{\underbrace{\;p(\pi_{t}|\pi_{t-1})}}\right]\left[\prod_{t=1}^{\tau }\underset{\text{emission}}{\underbrace{\;p(x_{t}|\pi_{t})}}\right],$$

where *K* is number of latent states, *τ* is the number of time steps and *L* the number of possible observed states. Parameters of the model are the initial state probability *π*_0_, transition probability *T* and emission probability *E*, defined as

4.2 $$\pi_{0}(i)=p(\pi_{1}=i),$$

4.3 $$T(i,j)=p(\pi_{t}=j|\pi_{t-1}=i)$$

4.4 $$\mathrm{and}\;\;\;\;E(i,j)=p(x_{t}=j|\pi_{t}=i).$$

Note that the rows of *T* and *E* are probability distributions by definition. We will jointly denote the model parameters as *θ* = {*π*_0_, *T*, *E*}. If *θ* is known, then we can estimate the latent states ***π*** conditional on parameters *θ* and observations **x** using the forward-filtering backward-sampling algorithm.

#### 4.2.2. Forward-filtering backward-sampling

Filtering is the estimation of the current hidden state by using all observations so far, *p*(*π*_*t*_ | ***x***_1:*t*_) [19]. This is known as *forward-filtering* and it can be represented as follows:

4.5 $$p(\pi_{t}|x_{1:t})\propto \sum_{\pi_{t-1}}p(\pi_{t},\pi_{t-1},x_{t}|x_{1:t-1})$$

4.6 $$=p(x_{t}|\pi_{t})\sum_{\pi_{t-1}}p(\pi_{t}|\pi_{t-1})p(\pi_{t-1}|x_{1:t-1}).$$

We define *α*(*π*_*t*_) = *p*(*π*_*t*_|**x**_1:*t*_) and substitute that into equation (4.6), which yields the following recursive equation known as **α*-recursion* or *forward recursion*;

4.7 $$\alpha (\pi_{t})\propto \underset{\text{corrector}}{\underbrace{\;p(x_{t}|\pi_{t})}}\underset{\text{predictor}}{\underbrace{\sum_{\pi_{t-1}}p(\pi_{t}|\pi_{t-1})\alpha (\pi_{t-1})}}$$

The recursion starts with *α*(*π*_1_) = *p*(*π*_1_|*x*_1_) = *p*(*x*_1_|*π*_1_)*p*(*π*_1_)/*p*(*x*_1_). This shows that the filtered distribution *α*(.) is propagated through to the next time step, where it acts like a ‘prior’. In other words, at each time step, the calculated posterior becomes the new prior for the next time step [27].

To estimate the posterior distribution of latent states, we sample from the joint distribution of the latent sequence, *p*(***π***_1:*τ*_|**x**_1:*τ*_)

4.8 $$p(\pi_{1:\tau }|x_{1:\tau })=p(\pi_{\tau }|x_{1:\tau })\prod_{t=1}^{\tau -1}p(\pi_{t}|\pi_{t+1},x_{1:t})$$

4.9 $$\propto p(\pi_{\tau }|x_{1:\tau })\prod_{t=1}^{\tau -1}p(\pi_{t+1}|\pi_{t})p(\pi_{t}|x_{1:t})$$

4.10 $$=\alpha (\pi_{\tau })\prod_{t=1}^{\tau -1}p(\pi_{t+1}|\pi_{t})\alpha (\pi_{t}).$$

According to equation (4.8), to be able to compute the posterior distribution of latent states, we need ‘time-reversed’ transitions *p*(*π*_*t*_|*π*_*t*+1_, ***x***_1:*t*_), which are obtained using **α*-recursion*s from the forward filtering. The simulation starts from the end of the sequence and proceeds backwards recursively. Starting point is

4.11 $${\hat{\pi }}_{\tau }∼\alpha (\pi_{\tau })=p(\pi_{\tau }|x_{1:\tau }).$$

For each time step *t*, the unnormalized sampling probability can be calculated using the previously sampled latent state $\pi_{t+1}={\hat{\pi }}_{t+1}$, and the ${\hat{\pi }}_{t}$ can be sampled using the following:

4.12 $${\hat{\pi }}_{t}∼p(\pi_{t+1}={\hat{\pi }}_{t+1}|\pi_{t})\alpha (\pi_{t}).$$

This procedure is known as forward-filtering backward-sampling [27].

#### 4.2.3. Learning model parameters

Conditionally on the latent states and observations the parameters *θ* = {*π*_0_, *T*, *E*} of the model can be estimated. The posterior distribution $p(\pi_{0},T,E|x_{1:\tau }^{1:N})$ is given by

4.13 $$\begin{array}{rl} p(\pi_{0},T,E & |x_{1:\tau }^{1:N},\pi_{1:\tau }^{1:N})\propto p(\pi_{0},T,E)p(x_{1:\tau }^{1:N},\pi_{1:\tau }^{1:N}\\ & |\pi_{0},T,E) \end{array}$$

4.14 $$\propto \underset{\text{prior}}{\underbrace{\;p(\pi_{0},T,E)}}\underset{\text{likelihood}}{\underbrace{\prod_{n=1}^{N}p(x_{1:\tau }^{n},\pi_{1:\tau }^{n}|\pi_{0},T,E).}}$$

Factorizing the likelihood term in equation (4.14) as in equation (4.1), we can get the following result:

4.15 $$\begin{array}{rl} \prod_{n=1}^{N}p(x_{1:\tau }^{n},\pi_{1:\tau }^{n} & |\pi_{0},T,E)=\prod_{n=1}^{N}\{\;p(\pi_{1}^{n}|\pi_{0})\prod_{t=2}^{\tau }p(\pi_{t}^{n}\\ & |\pi_{t-1}^{n},T)\prod_{t=1}^{\tau }p(x_{t}^{n}|\pi_{t}^{n},E)\} \end{array}$$

4.16 $$\begin{array}{rl} & =\left[\prod_{k=1}^{K}\pi_{0}{\left(k\right)}^{\pi_{0_{k}}^{\text{counts}}}\right]\left[\prod_{k=1}^{K}\prod_{l=1}^{K}T{\left(k,l\right)}^{T_{k,l}^{\text{counts}}}\right]\\ & \;\times \left[\prod_{k=1}^{K}\prod_{l=1}^{L}E{\left(k,l\right)}^{E_{k,l}^{\text{counts}}}\right]. \end{array}$$

Since, *π*_0_, rows of the *T*, and rows of the *E* are probability distribution, [.]’s inside equation (4.16) corresponds to multinomial distribution with parameters $\pi_{0}^{\text{counts}},T_{k,:}^{\text{counts}}$ and $E_{k,:}^{\text{counts}}$, where $\pi_{0}^{\text{counts}}$ corresponds to the initial state counts, *T*^counts^ corresponds to the state to state transition counts, and *E*^counts^ corresponds to the state to observation emission counts. Since the Dirichlet is conjugate prior for the multinomial distribution, we set a Dirichlet prior for *θ* = {*π*_0_, *T*, *E*} with hyperparameters $\pi_{0}^{\text{prior}},T_{k,:}^{\text{prior}}$ and $E_{k,:}^{\text{prior}}$, respectively. Because of the conjugacy, the resulting posterior in equation (4.14) is also a Dirichlet distribution

4.17 $$p(\pi_{0}|\pi_{0}^{\text{counts}},\pi_{0}^{\text{prior}})=\mathrm{Dirichlet}\;(\pi_{0}^{\text{counts}}+\pi_{0}^{\text{prior}}),$$

4.18 $$p(T_{k,:}|T_{k,:}^{\text{counts}},T_{k,:}^{\text{prior}})=\mathrm{Dirichlet}\;(T_{k,:}^{\text{counts}}+T_{k,:}^{\text{prior}})$$

4.19 $$\mathrm{and}\;p(E_{k,:}|E_{k,:}^{\text{counts}},E_{k,:}^{\text{prior}})=\mathrm{Dirichlet}\;(E_{k,:}^{\text{counts}}+E_{k,:}^{\text{prior}}).$$

#### 4.2.4. Inference of HMM with MCMC Sampling

There are several ways to estimate the parameters of HMM when both hidden states and model parameters *θ* are unknown, for example the expectation-maximization algorithm, variational Bayes, or MCMC sampling. In this paper, we will use MCMC. The goal of MCMC is to produce draws from the posterior *p*(***π***, *θ*|**x**). The Gibbs sampler is an MCMC algorithm suitable for high-dimensional problems, such as the HMM, and it samples parameters one-by-one using their distributions conditional on the other parameters. In the HMM, it will alternate between sampling the model parameters *θ* conditional on the latent sequence ***π*** from *p*(*θ*|***π***, **x**), and the latent sequence ***π*** conditional on the model parameters *θ* from *p*(***π***|*θ*, **x**). The algorithm iterates the following steps *N* times, which gives *N* draws from the posterior:

1. Sample the model parameters $\theta^{*}=\{\pi_{0}^{*},T^{*},E^{*}\}$ from equations (4.17), (4.18) and (4.19), respectively. (Note that they are independent of each other given latent sequence.)
2. Sample the latent sequence of HMM using the updated model parameters *θ* using the forward-filtering backward-sampling algorithm.

**Figure 7.**
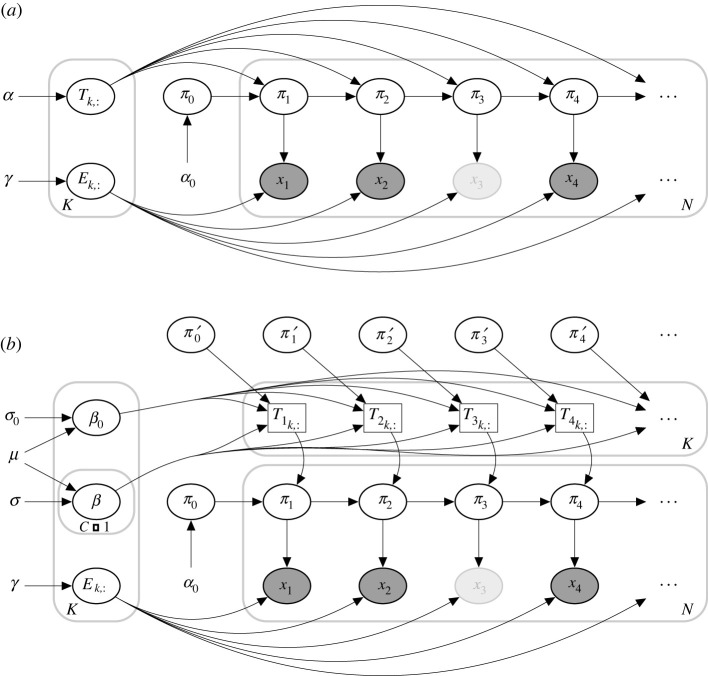
Plate diagram of (*a*) hidden Markov model (HMM) and (*b*) coupled hidden Markov model (CHMM). Here, for clarity, we illustrate only one chain. *π*_*t*_ represents the unobserved true states, and *x*_*t*_ represents the noisy observations at time *t*. The faded observation nodes correspond to missing values. Plain parameters are hyperparameters of the model. Edges from *π*_*t*_ to *x*_*t*_ represent *emission probabilities*. In the CHMM, the next state in one site depends on the previous states of the other sites. It is illustrated by *π*^′^. Therefore, *transition probabilities* change at each time step.

Before calculating the posteriors, the first *N*_0_ samples are discarded as a convergence (warm-up) period of the Markov chain. The plate diagram of the HMM is given in figure 7*a*, and the algorithm is described in algorithm 1.

**Algorithm 1.** Hidden Markov Model.

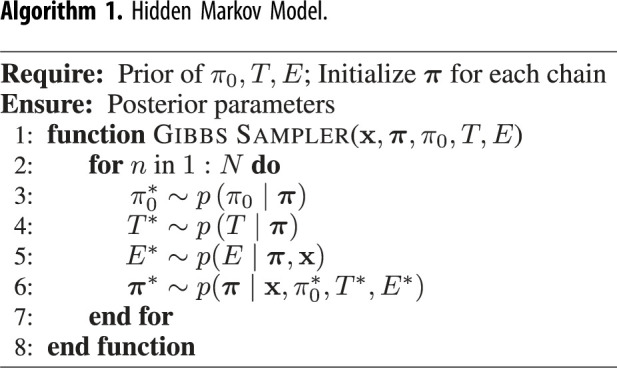

#### 4.2.5. Coupled hidden Markov model

In the set of ordinary HMMs, transition parameters do not change within the chain. However, in the CHMM, transitions in one chain are affected by other chains. In principle, it would be possible to define a single joint HMM where the latent state represents the latent states of all individual chains jointly and adapt the solution for the HMM described above. However, this is inefficient when there are many chains because the number of states would grow as *O*(*K*^*C*^), where *K* is the number of hidden states in a chain and *C* is the number of chains. On the high level, our key idea is that the transition matrix of each chain is modelled conditionally on the states of the other chains (and not as a single large joint transition matrix). A similar but simpler formulation was considered by [12]. Note that a transition matrix *T* for a specific chain will not be time-independent any more, instead, changes at each time step depend on the states of the other chains. The graphical representation of the model (showing just two chains) is given in figure 1, and the plate diagram of the CHMM is given in figure 7*b*. There are *O*(*KC*) parameters in this formulation, which is much more efficient for the increased number of chains. In theory, CHMM is a low-rank estimation of one joint HMM.

Defining the transition matrix is a critical part of the algorithm, and it is essential to carry as much information about the other chains as possible. We model the dependencies between the chains with parameters ***β***, where each *β*_[__.]_ is a matrix of the same size with the transition matrix. Assume there are *C* chains and let C denote the set of chains. Further, assume that each chain can be in one of *K* possible states, and let K denote the set of states. Finally, assume that there is a baseline state $\hat{k}$ such that if a chain is in that state, it does not affect other chains (in our application, this state corresponds to the absence of MRSA colonization in the respective body site). The transition matrix for the chain $\hat{c}$, at time *t*, denoted as $T_{t}^{\left[\hat{c}\right]}$, is defined by

4.20 $$U_{t}^{\left[\hat{c}\right]}=\beta_{0}^{\left[\hat{c}\right]}+\sum_{c\in \{C\setminus \hat{c}\}}\sum_{k\in \{K\setminus \hat{k}\}}\beta_{k}^{\left[\hat{c}\leftarrow c\right]}I[\pi_{t-1}^{\left[c\right]}=k]$$

and

4.21 $$T_{t}^{\left[\hat{c}\right]}=\sigma_{\text{row}}(U_{t}^{\left[\hat{c}\right]}).$$

The transition matrix $T_{t}^{\left[\hat{c}\right]}$ is obtained in equation (4.21) by applying a row-wise softmax operator *σ*_row_ to the unnormalized transition matrix $U_{t}^{\left[\hat{c}\right]}$. Parameter $\beta_{0}^{\left[\hat{c}\right]}$ corresponds to an intercept matrix and it specifies the transition matrix of the target chain $\hat{c}$ when all other chains are in the baseline state $\hat{k}$. Parameter $\beta_{k}^{\left[\hat{c}\leftarrow c\right]}$ represents the impact of chain *c* on the target chain $\hat{c}$ and it is added to $\beta_{0}^{\left[\hat{c}\right]}$ whenever chain *c* was in state $k\neq \hat{k}$ in the previous time step. We will denote all the parameters for target chain $\hat{c}$ as $\beta^{\left[\hat{c}\right]}$.

In equation (4.20), the unnormalized transition probability *U* is an additive function of the latent states of the other chains; hence we call it the *Additive-CHMM*. We also implemented a simpler formulation than the Additive-CHMM, which we call *Or-CHMM*. In the Or-CHMM, the target chain is not affected by the other chains individually; instead, there the target chain is affected whenever *any* of the other chains is in some state different from the baseline state, and the unnormalized transition matrix is given by

4.22 $$U_{t}^{\left[\hat{c}\right]}=\beta_{0}^{\left[\hat{c}\right]}+\sum_{k\in \{K\setminus \hat{k}\}}\beta_{k}^{\left[\hat{c}\right]}I[\exists c\in \{C\setminus \hat{c}\}:\pi_{t-1}^{\left[c\right]}=k].$$

In our application, we have *K* = 2, corresponding to the presence and absence of colonization in a given body site. In this case, equation (4.22) has a simple form: if all other chains are in state $\hat{k}$, the output is only $\beta_{0}^{\left[\hat{c}\right]}$, and if any of the other chains is in a state other $k\neq \hat{k}$, then the output is the sum of $\beta_{0}^{\left[\hat{c}\right]}$ and $\beta_{k}^{\left[\hat{c}\right]}$.

#### 4.2.6. Design and interpretation of the *β* parameters

In the CHMM, the transition matrix *T* for each chain is modelled as a function of *β* parameters, where each *β*_*k*_ is a matrix of the same size as the transition matrix. Therefore, the *β* parameters are a list of matrices. Since the rows of a transition matrix *T* are assumed independent, we also model *β* parameters such that the rows of those matrices are independent. However, to avoid redundant parameters, the entries on each row of *β* are assumed to sum to zero. In practice, we sample the first *K* − 1 parameter on a given row and set the *K*th element to equal the negative of the sum of all the other parameters. This constraint ensures that the mapping from *β* to *T* is one-to-one.

To give an interpretation to the parameters *β* in the Additive-CHMM, we start with equation (4.21) and write the softmax for a single element of a transition matrix *T*(*i*, *j*)

4.23 $$T_{t}(i,j)=\frac{\exp(U_{t}(i,j))}{\exp(U_{t}(i,j))+\sum_{l\in \{K\setminus k\}}\exp(U_{t}(i,l))},$$

from which it follows after straightforward algebra

4.24 $$\text{logit}(T_{t}(i,j))=U_{t}(i,j)-\log\left(\sum_{l\in \{K\setminus k\}}\exp(U_{t}(i,j))\right).$$

In our application *K* = 2 and sums of the rows of *β*_*k*_ are assumed equal to 0, so consequently also the rows in the *U*_*t*_ sum to zero, which gives us

4.25 $$\;\text{logit}(T_{t}(i,j))=U_{t}(i,j)-U_{t}(i,\neg j)$$

4.26 $$=2U_{t}(i,j),$$

where ¬j refers to the other element on the row that is not *j*. Therefore, in this two-dimensional case, all *β*_0_ values correspond to half of the log-odds of the respective transition probability, and similarly, parameters *β*_*k*_ representing the interactions between the chains correspond to half of the change in the log-odds because of the presence of colonization in the other chain.

#### 4.2.7. Adaptive Metropolis–Hastings within Gibbs sampling

So far, we have described the design specifications of the CHMM. Since transitions are changing at each time step, it is not feasible to calculate their sufficient statistics as in equation (4.16). To resolve this, we sample the $\beta^{[\hat{c}]*}$ using a Metropolis–Hastings (MH) step within the Gibbs sampler because conditional on the latent states of all the chains, the transition parameters are fully determined by the $\beta^{\left[\hat{c}\right]}$ parameters. To get a draw from $p(\beta^{\left[\hat{c}\right]}|\pi_{1:\tau }^{\left[\hat{c}\right]},\pi_{1:\tau }^{\left[-\hat{c}\right]})$, where $\pi_{1:\tau }^{\left[-\hat{c}\right]}$ are states of the other chains, we use the following steps:

1. Make a proposal $\beta^{[\hat{c}]*}∼q(\beta^{\left[\hat{c}\right]})$
2. Transform $\beta^{[\hat{c}]*}$ samples to transition probabilities $T_{1:\tau }^{[\hat{c}]*}$ according to latent states of the other chains, *π*^′^
3. Accept the proposal $\beta^{[\hat{c}]*}$ with probability
  4.27 $$\alpha (T_{1:\tau }^{[\hat{c}]*})=\min\left\{1,\frac{\;p(\beta^{[\hat{c}]*}|\pi_{1:\tau }^{\left[\hat{c}\right]},\pi_{1:\tau }^{\left[-\hat{c}\right]})q(\beta^{\left[\hat{c}\right]}|\beta^{[\hat{c}]*})}{p(\beta^{\left[\hat{c}\right]}|\pi_{1:\tau }^{\left[\hat{c}\right]},\pi_{1:\tau }^{\left[-\hat{c}\right]})q(\beta^{[\hat{c}]*}|\beta^{\left[\hat{c}\right]})}\right\},$$
  where
  4.28 $$p(\beta^{\left[\hat{c}\right]}|\pi_{1:\tau }^{\left[\hat{c}\right]},\pi_{1:\tau }^{\left[-\hat{c}\right]})\propto p(\pi_{1:\tau }^{\left[\hat{c}\right]}|\beta^{[\hat{c}]*},\pi_{1:\tau }^{\left[\hat{c}\right]})p(\beta^{[\hat{c}]*}).$$

The first quantity on the right-hand side of equation (4.28) can be directly calculated with the transition parameters $T_{1:\tau }^{[\hat{c}]*}$ for corresponding chain. We used the Gaussian proposal in which the previously sampled $\beta^{\left[\hat{c}\right]}$ is the mean such that $q(\beta^{[\hat{c}]*}|\beta^{\left[\hat{c}\right]})=N(\beta^{[\hat{c}]*}|\beta^{\left[\hat{c}\right]},c^{2}\Sigma )$. As a variance parameter, we used a fixed diagonal covariance matrix during the warm-up period and then used the covariance matrix estimated from the previous samples. We initially set $c\approx 2.38/\sqrt{d}$ as Metropolis jumping scaling factor since it is theoretically the most efficient scaling factor [28], where *d* is the dimension of the sampling. Then we adaptively scaled the scaling factor *c* as described in [29]. Since this proposal distribution is symmetric (i.e. normal), $q(\beta^{\left[\hat{c}\right]}|\beta^{[\hat{c}]*})$ and $q(\beta^{[\hat{c}]*}|\beta^{\left[\hat{c}\right]})$ in equation (4.27), cancel out each other.

To sample the latent sequences, we need to modify the forward-filtering backward-sampling algorithm [15], since we need to draw samples from $p(\pi_{1:\tau }^{\left[\hat{c}\right]}|\pi_{1:\tau }^{\left[-\hat{c}\right]},x_{1:\tau }^{\left[\hat{c}\right]})$ instead of $p(\pi_{1:\tau }^{\left[\hat{c}\right]}|x_{1:\tau }^{\left[\hat{c}\right]})$. The modified *forward-filtering* is defined as follows:

4.29 $$\begin{array}{rl} p(\pi_{t}^{\left[\hat{c}\right]} & |\pi_{1:t+1}^{\left[-\hat{c}\right]},x_{1:t}^{\left[\hat{c}\right]})=\sum_{\pi_{t-1}^{\left[\hat{c}\right]}}p(\pi_{t}^{\left[\hat{c}\right]},\pi_{t-1}^{\left[\hat{c}\right]}\\ & |x_{1:t-1}^{\left[\hat{c}\right]},x_{t}^{\left[\hat{c}\right]},\pi_{1:t}^{\left[-\hat{c}\right]},\pi_{t+1}^{\left[-\hat{c}\right]}) \end{array}$$

4.30 $$\begin{array}{rl} \propto p(x_{t}^{\left[\hat{c}\right]} & |\pi_{t}^{\left[\hat{c}\right]})\prod_{c^{\prime }\neq \hat{c}}p(\pi_{t+1}^{\left[c^{\prime }\right]}|\pi_{t}^{\left[c^{\prime }\right]},\pi_{t}^{\left[-c^{\prime }\right]})\sum_{\pi_{t-1}^{\left[\hat{c}\right]}}p(\pi_{t}^{\left[\hat{c}\right]}\\ & |\pi_{t-1}^{\left[\hat{c}\right]},\pi_{t-1}^{\left[-\hat{c}\right]})p(\pi_{t-1}^{\left[\hat{c}\right]}|\pi_{1:t}^{\left[-\hat{c}\right]},x_{1:t-1}^{\left[\hat{c}\right]}). \end{array}$$

If we define $\alpha (\pi_{t}^{\left[\hat{c}\right]})=p(\pi_{t}^{\left[\hat{c}\right]}|\pi_{1:t+1}^{\left[-\hat{c}\right]},x_{1:t}^{\left[\hat{c}\right]})$ and substitute that into equation (4.30), we can acquire *modified *α*-recursion*;

4.31 $$\alpha (\pi_{t}^{\left[\hat{c}\right]})\propto \underset{\text{corrector}}{\underbrace{\;p(x_{t}^{\left[\hat{c}\right]}|\pi_{t}^{\left[\hat{c}\right]})}}\underset{\text{modifying mass}}{\underbrace{\prod_{c^{\prime }\neq \hat{c}}p(\pi_{t+1}^{\left[c^{\prime }\right]}|\pi_{t}^{\left[c^{\prime }\right]},\pi_{t}^{\left[-c^{\prime }\right]})}}\underset{\text{predictor}}{\underbrace{\sum_{\pi_{t-1}^{\left[\hat{c}\right]}}p(\pi_{t}^{\left[\hat{c}\right]}|\pi_{t-1}^{\left[\hat{c}\right]},\pi_{t-1}^{\left[-\hat{c}\right]})\alpha (\pi_{t-1}^{\left[\hat{c}\right]})}}$$

Respectively, the posterior distribution of the latent sequences for each chain can be acquired as follows:

4.32 $$\begin{array}{rl} p(\pi_{1:\tau }^{\left[\hat{c}\right]} & |\pi_{1:\tau }^{\left[-\hat{c}\right]},x_{1:\tau }^{\left[\hat{c}\right]})=p(\pi_{\tau }^{\left[\hat{c}\right]}|\pi_{1:\tau }^{\left[-\hat{c}\right]},x_{1:\tau }^{\left[\hat{c}\right]})\prod_{t=1}^{\tau -1}p(\pi_{t}^{\left[\hat{c}\right]}\\ & |\pi_{t+1}^{\left[\hat{c}\right]},\pi_{1:t+1}^{\left[-\hat{c}\right]},x_{1:t}^{\left[\hat{c}\right]}) \end{array}$$

4.33 $$\begin{array}{rl} \propto p(\pi_{\tau }^{\left[\hat{c}\right]} & |\pi_{1:\tau }^{\left[-\hat{c}\right]},x_{1:\tau }^{\left[\hat{c}\right]})\prod_{t=1}^{\tau -1}p(\pi_{t+1}^{\left[\hat{c}\right]}|\pi_{t}^{\left[\hat{c}\right]},\pi_{t}^{\left[-\hat{c}\right]})p(\pi_{t}^{\left[\hat{c}\right]}\\ & |\pi_{1:t+1}^{\left[-\hat{c}\right]},x_{1:t}^{\left[\hat{c}\right]}) \end{array}$$

4.34 $$=\alpha (\pi_{\tau }^{\left[\hat{c}\right]})\prod_{t=1}^{\tau -1}p(\pi_{t+1}^{\left[\hat{c}\right]}|\pi_{t}^{\left[\hat{c}\right]},\pi_{t}^{\left[-\hat{c}\right]})\alpha (\pi_{t}^{\left[\hat{c}\right]}).$$

Therefore, we can use the sampling procedure described in equations (4.11) and (4.12). Algorithm 2 summarizes the whole algorithm. To validate the implementation, we used the algorithm to estimate parameters in simulated data. The results in electronic supplementary material, figure S2 show that the algorithm estimated the parameters correctly and yielded well-calibrated posterior distributions.

**Algorithm 2.** Coupled Hidden Markov Model.

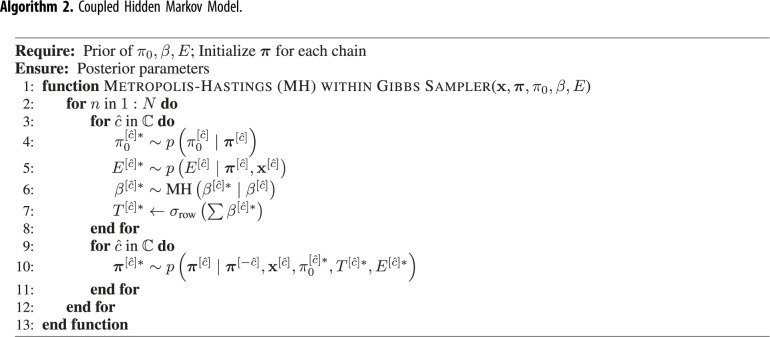

#### 4.2.8. Implementation details

We set the initial covariance matrix for the Metropolis proposal as 0.01 × *I*, where *I* is the identity matrix, which corresponds to a step size giving the optimal acceptance rate of approximately 23% [30]. We set the prior of *β*_0_ as *N*(*β*_0_| 0, 1), which is almost uninformative so that the estimates are not affected strongly by the prior. For the rest of the *β* parameters, denoted by *β*_*k*_, we used sparsity encouraging Horseshoe prior with mean and scale parameters of 0 and 0.25, respectively (see electronic supplementary material). We used a uniform prior on the initial state probabilities *π*_0_, and weak Dirichlet priors for the rows of emission probabilities *E* such that we set the value to 30 for specificity and 15 for sensitivity. We set the rest of emission priors to 1, which corresponds to uniform prior. In such a formulation, except for the initialization, the prior has a negligible effect because it is summed with observation counts during inference. We drew 50 000 MCMC samples, and we set the warm-up length as 25 000. Posterior probabilities are calculated using the remaining MCMC samples. HMM and CHMM implicitly assume that time intervals between observations are the same, which is not the case in our data. Therefore, during the model training, we assumed that there are missing observations at two, four and five months after enrolment.

## Data Availability

The CLEAR (Changing Lives by Eradicating Antibiotic Resistance) trial demonstrated that the use of a post-discharge decolonization protocol in MRSA carriers reduces infection and hospitalization rates; ClinicalTrials.gov no. NCT01209234, see [9] for informed consent and institutional review board approvals. Code accessibility: the R package is accessible at https://github.com/onurpoyraz/chmmMCMC. The data are provided in electronic supplementary material [31].

## References

[1] Gorwitz RJ et al. 2008 Changes in the prevalence of nasal colonization with *Staphylococcus aureus* in the United States, 2001–2004. J. Infect. Dis. **197**, 1226-1234. (10.1086/533494) 18422434

[2] Weiner-Lastinger LM et al. 2020 Antimicrobial-resistant pathogens associated with adult healthcare-associated infections: summary of data reported to the National Healthcare Safety Network, 2015–2017. Infect. Control Hosp. Epidemiol. **41**, 1-18. (10.1017/ice.2019.296) 31767041 PMC8276252

[3] Centers for Disease Control and Prevention US. 2019 *Antibiotic resistance threats in the United States, 2019*. US Department of Health and Human Services, Centers for Disease Control and Prevention. (10.15620/cdc:82532)

[4] Kourtis AP et al. 2019 Vital signs: epidemiology and recent trends in methicillin-resistant and in methicillin-susceptible *Staphylococcus aureus* bloodstream infections–United States. Morb. Mortal. Wkly Rep. **68**, 214. (10.15585/mmwr.mm6809e1) PMC6421967 30845118

[5] Johnson PD et al. 2005 Efficacy of an alcohol/chlorhexidine hand hygiene program in a hospital with high rates of nosocomial methicillin-resistant *Staphylococcus aureus* (MRSA) infection. Med. J. Aust. **183**, 509-514. (10.5694/j.1326-5377.2005.tb07151.x) 16296963

[6] Kluytmans J, Van Belkum A, Verbrugh H. 1997 Nasal carriage of *Staphylococcus aureus*: epidemiology, underlying mechanisms, and associated risks. Clin. Microbiol. Rev. **10**, 505-520. (10.1128/CMR.10.3.505) 9227864 PMC172932

[7] McKinnell JA, Huang SS, Eells SJ, Cui E, Miller LG. 2013 Quantifying the impact of extranasal testing of body sites for methicillin-resistant *Staphylococcus aureus* colonization at the time of hospital or intensive care unit admission. Infect. Control Hosp. Epidemiol. **34**, 161-170. (10.1086/669095) 23295562 PMC3894230

[8] Perl TM et al. 2002 Intranasal mupirocin to prevent postoperative *Staphylococcus aureus* infections. N. Engl. J. Med. **346**, 1871-1877. (10.1056/NEJMoa003069) 12063371

[9] Huang SS et al. 2019 Decolonization to reduce postdischarge infection risk among MRSA carriers. N. Engl. J. Med. **380**, 638-650. (10.1056/NEJMoa1716771) 30763195 PMC6475519

[10] Brand M, Oliver N, Pentland A. 1997 Coupled hidden Markov models for complex action recognition. In *Proc. of IEEE Computer Society Conf. on Computer Vision and Pattern Recognition*, pp. 994–999. IEEE. (10.1109/CVPR.1997.609450)

[11] Brand M. 1997 Coupled hidden Markov models for modeling interacting processes. *Technical report*.

[12] Sherlock C, Xifara T, Telfer S, Begon M. 2013 A coupled hidden Markov model for disease interactions. J. R. Stat. Soc.: Ser. C (Appl. Stat.) **62**, 609-627. (10.1111/rssc.12015) PMC3813975 24223436

[13] Rezek I, Sykacek P, Roberts SJ. 2000 Learning interaction dynamics with coupled hidden Markov models. IEE Proc.-Sci., Meas. Technol. **147**, 345-350. (10.1049/ip-smt:20000851)

[14] Zhong S, Ghosh J. 2001 *A new formulation of coupled hidden Markov models*. Austin, TX: The University of Texas Department of Electrical and Computer Engineering.

[15] Touloupou P, Finkenstädt B, Spencer SE. 2020 Scalable Bayesian inference for coupled hidden Markov and semi-Markov models. J. Comput. Graph. Stat. **29**, 238-249. (10.1080/10618600.2019.1654880) 32939192 PMC7455056

[16] Ghahjaverestan NM et al. 2015 Coupled hidden Markov model-based method for *apnea bradycardia* detection. IEEE J. Biomed. Health Inform. **20**, 527-538. (10.1109/JBHI.2015.2405075) 25706937

[17] Rabiner LR. 1989 A tutorial on hidden Markov models and selected applications in speech recognition. Proc. IEEE **77**, 257-286. (10.1109/5.18626)

[18] Murphy KP. 2012 Machine learning: a probabilistic perspective. Cambridge, MA: MIT Press.

[19] Barber D, Cemgil AT. 2010 Graphical models for time-series. IEEE Signal Process Mag. **27**, 18-28. (10.1109/MSP.2010.938028)

[20] Gelman A, Hwang J, Vehtari A. 2014 Understanding predictive information criteria for Bayesian models. Stat. Comput. **24**, 997-1016. (10.1007/s11222-013-9416-2)

[21] Von Eiff C, Becker K, Machka K, Stammer H, Peters G. 2001 Nasal carriage as a source of *Staphylococcus aureus* bacteremia. N. Engl. J. Med. **344**, 11-16. (10.1056/NEJM200101043440102) 11136954

[22] Hall MD et al. 2019 Improved characterisation of MRSA transmission using within-host bacterial sequence diversity. Elife **8**, e46402. (10.7554/eLife.46402) 31591959 PMC6954020

[23] Cepeda JA et al. 2005 Isolation of patients in single rooms or cohorts to reduce spread of MRSA in intensive-care units: prospective two centre study. The Lancet **365**, 295-304. (10.1016/S0140-6736(05)17783-6) 15664224

[24] Huskins WC et al. 2011 Intervention to reduce transmission of resistant bacteria in intensive care. N. Engl. J. Med. **364**, 1407-1418. (10.1056/NEJMoa1000373) 21488763 PMC3410743

[25] Carr AL, Daley MJ, Givens Merkel K, Rose DT. 2018 Clinical utility of methicillin-resistant *Staphylococcus aureus* nasal screening for antimicrobial stewardship: a review of current literature. Pharmacother.: J. Hum. Pharmacol. Drug Ther. **38**, 1216-1228. (10.1002/phar.2188) 30300441

[26] Datta R et al. 2014 High nasal burden of methicillin-resistant *Staphylococcus aureus* increases risk of invasive disease. J. Clin. Microbiol. **52**, 312-314. (10.1128/JCM.01606-13) 24153126 PMC3911445

[27] Barber D. 2012 Bayesian reasoning and machine learning. Cambridge, UK: Cambridge University Press.

[28] Gelman A. 2013 Bayesian data analysis. Boca Raton, FL: CRC Press

[29] Sherlock C, Fearnhead P, Roberts GO. 2010 The random walk Metropolis: linking theory and practice through a case study. Stat. Sci. **25**, 172-190. (10.1214/10-STS327)

[30] Roberts GO, Rosenthal JS. 2001 Optimal scaling for various Metropolis-Hastings algorithms. Stat. Sci. **16**, 351-367. (10.1214/ss/1015346320)

[31] Poyraz O, Sater MRA, Miller LG, McKinnell JA, Huang SS, Grad YH, Marttinen P. 2022 Modelling methicillin-resistant *Staphylococcus aureus* decolonization: interactions between body sites and the impact of site-specific clearance. Figshare. (10.6084/m9.figshare.c.6016851) PMC9198502 35702866

